# Double Strand Breaks Can Initiate Gene Silencing and SIRT1-Dependent Onset of DNA Methylation in an Exogenous Promoter CpG Island

**DOI:** 10.1371/journal.pgen.1000155

**Published:** 2008-08-15

**Authors:** Heather M. O'Hagan, Helai P. Mohammad, Stephen B. Baylin

**Affiliations:** The Sidney Kimmel Comprehensive Cancer Center at Johns Hopkins, Baltimore, Maryland, United States of America; Massachusetts General Hospital, Howard Hughes Medical Institute, United States of America

## Abstract

Chronic exposure to inducers of DNA base oxidation and single and double strand breaks contribute to tumorigenesis. In addition to the genetic changes caused by this DNA damage, such tumors often contain epigenetically silenced genes with aberrant promoter region CpG island DNA hypermethylation. We herein explore the relationships between such DNA damage and epigenetic gene silencing using an experimental model in which we induce a defined double strand break in an exogenous promoter construct of the E-cadherin CpG island, which is frequently aberrantly DNA hypermethylated in epithelial cancers. Following the onset of repair of the break, we observe recruitment to the site of damage of key proteins involved in establishing and maintaining transcriptional repression, namely SIRT1, EZH2, DNMT1, and DNMT3B, and the appearance of the silencing histone modifications, hypoacetyl H4K16, H3K9me2 and me3, and H3K27me3. Although in most cells selected after the break, DNA repair occurs faithfully with preservation of activity of the promoter, a small percentage of the plated cells demonstrate induction of heritable silencing. The chromatin around the break site in such a silent clone is enriched for most of the above silent chromatin proteins and histone marks, and the region harbors the appearance of increasing DNA methylation in the CpG island of the promoter. During the acute break, SIRT1 appears to be required for the transient recruitment of DNMT3B and subsequent methylation of the promoter in the silent clones. Taken together, our data suggest that normal repair of a DNA break can occasionally cause heritable silencing of a CpG island–containing promoter by recruitment of proteins involved in silencing. Furthermore, with contribution of the stress-related protein SIRT1, the break can lead to the onset of aberrant CpG island DNA methylation, which is frequently associated with tight gene silencing in cancer.

## Author Summary

Human cancers contain epigenetic changes as well as DNA mutations that play a role in abnormal silencing of tumor suppressor genes. In contrast to DNA mutations that change the sequence of DNA, epigenetic changes cause abnormal silencing of genes through DNA methylation via the addition of methyl groups to DNA and through modifications to the associated chromatin proteins. One important event in tumor initiation and progression is the exposure of cells to DNA damage during events such as chronic inflammation and carcinogen exposure. We hypothesized that such damage may play a role in producing chromatin alterations, which could initiate epigenetic silencing of tumor suppressor genes. Here we show, using an exogenous gene promoter model, that key proteins involved in epigenetic silencing are recruited to the DNA near a double strand break. Occasionally, sustained localization of these proteins to the gene promoter leads to silencing of the associated gene and to the seeding and spreading of DNA methylation within the promoter that further stabilizes the silencing. This finding suggests that DNA damage may directly contribute to the large number of epigenetically silenced genes in tumors.

## Introduction

Chronic inflammation along with aging causes an increase in reactive oxygen species that induces DNA damage in the form of base oxidation, single stand breaks, and double strand breaks (DSBs) [1]. Errors in DSB repair can cause mutations and chromosome instability that lead to cancer or cell death [2]. In response to DSBs, cells undergo cell cycle arrest or apoptosis. Cell cycle arrest gives the cell time to repair the damage utilizing repair proteins that are recruited to the site of damage and activated. DSBs are repaired by either homologous recombination (HR) or nonhomologous end joining (NHEJ) [3]. The pathway followed to repair DSBs is determined by the location in the cell cycle and the type of cell [4].

The above repair processes occur in DNA that is often packaged in highly organized, mostly condensed chromatin, which also consists of histones and histone-associated proteins. Chromatin structure and dynamics regulate the genome such that non-desirable transcription is repressed [5]. This chromatin structure is determined by modifications of histone tails by acetylation, methylation, and phosphorylation in patterns which have been termed the histone code [6]. In general, acetylation of lysine residues induces an open chromatin configuration associated with gene expression, whereas deacetylation induces closed, compact chromatin associated with transcriptional repression. The amino-terminal tails of both histones H3 and H4 contain several lysine residues that can be acetylated by histone acetyl transferases (HATs) and deacetylated by histone deacetylases (HDACs) [7],[8]. Acetylation neutralizes the positive charge of the lysine residues and changes the structure of the histone, likely affecting the interaction of these histones with both proteins and DNA [9]. Specifically, mutational studies have indicated that lysine 16 of histone H4 (H4K16) and lysines 9, 14, and 18 of H3 are critical in silencing and are all acetylated in active chromatin and hypoacetlyated in transcriptionally-repressive chromatin [9],[10]. Histone methylation also plays a role in chromatin dynamics with mono-, di-, and tri-methylation of H3K4 being associated with active chromatin, and alternatively with mono-, di-, and tri-methylation of H3K9 and H4K20 and di- and tri-methylation of H3K27 being associated with closed chromatin and gene silencing [11]–[13].

It has become increasingly apparent that DNA repair must be intimately involved with regulation of chromatin. For the repair of DSBs there is an access, repair, restore (ARR) hypothesis wherein chromatin after a DSB is first modified to generate an open chromatin structure allowing access to the DNA by repair proteins [14]. Additionally, specific modifications of chromatin may be necessary for components of the DNA repair or checkpoint machinery to recognize damaged DNA [15]. In support of this step, in yeast, both HATs and chromatin remodeling complexes are recruited to DSBs [16]–[19]. Repair of the break then occurs, followed by the need to restore the chromatin back to its original, more condensed state [14],[20]. Condensed chromatin might also prevent transcription and/or replication machinery from accessing the DNA and/or interfering with the repair process [21],[22]. Additionally, condensed chromatin may play a role in ending the DNA damage response signaling cascade [23]. Restoration of the chromatin around a break suggests that silencing factors such as HDACs and histone methyl transferases (HMTs) might be recruited to the area of DSBs [19]. Additionally, DNA methylation patterns also need to be restored, suggesting a possible role for DNA methyltransferases (DNMTs) in DSB repair [24].

One example of a histone-modifying protein involved in DNA repair in the yeast *S. cervisiae* is Sir2, a NAD+ dependent protein and histone deacetylase [25]. The family of yeast sirtuins (Sir2-4) has been shown to be involved in telomeric silencing, silencing at the mating-type locus, and DSB repair [26],[27]. In telomeres Sir2-4, with the help of Rap1, a telomere DNA-binding protein, polymerize across nucleosomes by binding to the histone tails of H3 and H4 to create an inactive heterochromatin state causing silencing of the region [28]. In response to activation of the DNA damage checkpoint pathway Sir2-4 are recruited from the telomeres to the DSB where they facilitate end-joining to such an extent that yeast with mutations in any of the Sir proteins have a defect in NHEJ [29],[30]. Sir2 specifically modifies chromatin by deacetylating H4K16 and H3K9 [31]. In the area of a defined DSB in yeast there is an increase followed by a decrease in H4K16 acetylation [19]. Localization of Sir2 to the region occurs in the same time frame as the decrease in acetylation, suggesting that Sir2 is responsible for the deacetylation [19]. Additionally, mutations of four lysine residues on the histone H4 tail increase the sensitivity of yeast to DSB-inducing agents [32]. In mammalian cells, acetylation of H4 also seems to play a role in DSB repair because TRRAP (Transactivation-transformation domain-associated protein)/TIP60 (HIV Tat-Interacting Protein, 60 kDa) dependent acetylation of H4 occurs immediately after a DSB [33]. TIP60 binds to the chromatin around the DSB and plays a role in chromatin relaxation required for the efficient recruitment of repair factors as well as repair of the DSB [33]. Mutants that lack this ability accumulate DSBs following exposure to gamma-irradiation [34]. After repair of the DSB, there may be a need to deacetylate H4 to return the acetylation levels back to normal.

SIRT1, the mammalian homolog of Sir2, mediates transcriptional repression, heterochromatin formation, heritable gene silencing, p53 function, and lifespan [35]–[39]. SIRT1 has been shown to be involved in the maintenance of silencing associated with abnormal promoter region CpG island DNA methylation in tumor suppressor genes [39]. SIRT1 is localized to the promoters of these methylated and silenced tumor suppressor genes, but not to promoters of the same genes in cell lines where they are normally maintained in an unmethylated, open chromatin state facilitating gene expression [39]. Inhibition of SIRT1 caused re-expression of these genes along with a corresponding increase in H4K16 and H3K9 acetylation and SIRT1 recruitment to an artificial promoter via a gal4 DNA binding site mediates transcriptional repression, H4K16 deacetylation, and an increase in H3K9me3 [40]. Additionally SIRT1 has been found in a stem/precursor cell and/or “transformation specific” polycomb group (PcG) complex (PRC4) containing Enhancer of Zeste Homologue 2 (EZH2), the enzyme catalyzing H3K27me3 and H1K26me [41],[42], and Eed2 [43]. Previously, EZH2 had been identified as part of the PRC2/3 complex that plays a role in the initiation of chromatin silencing during development [41],[42]. SIRT1 is also linked to the increased methylation of H3K9 because SIRT1 has been shown to bind to, and increase the activity of, the suppressor of variegation 3–9 homologue (SUV39H1), a HMT that tri-methylates H3K9 [44]. These findings suggest that the recruitment of SIRT1 (and possibly EZH2) to the promoter of a gene can induce gene silencing via closed chromatin and that the continual presence of SIRT1 helps maintain the silencing.

In this study, we demonstrate the recruitment of silencing factors to a DSB induced in a model exogenous construct containing the CpG island region of the E-cadherin (E-cad) promoter, which is often aberrantly silenced and DNA hypermethylated in human cancer [45]. After an induced break, both SIRT1 and EZH2 are transiently recruited to the area surrounding the break. Their recruitment corresponds, following an initial increase, to a decrease in H4K16ac and an increase in H3K27me3. Additionally, DNMT1 and DNMT3B are also transiently recruited to the break site. By inducing DNA damage and then selecting for silencing of the HSVTK gene, driven by the E-cad promoter in our system, we demonstrate occasional gene silencing and onset of DNA methylation in the CpG island area. Moreover, the induced DNA methylation and recruitment of DNMT3B appear to be dependent on the presence of SIRT1 during the initial break and repair cycle.

## Results

**Figure 1:**
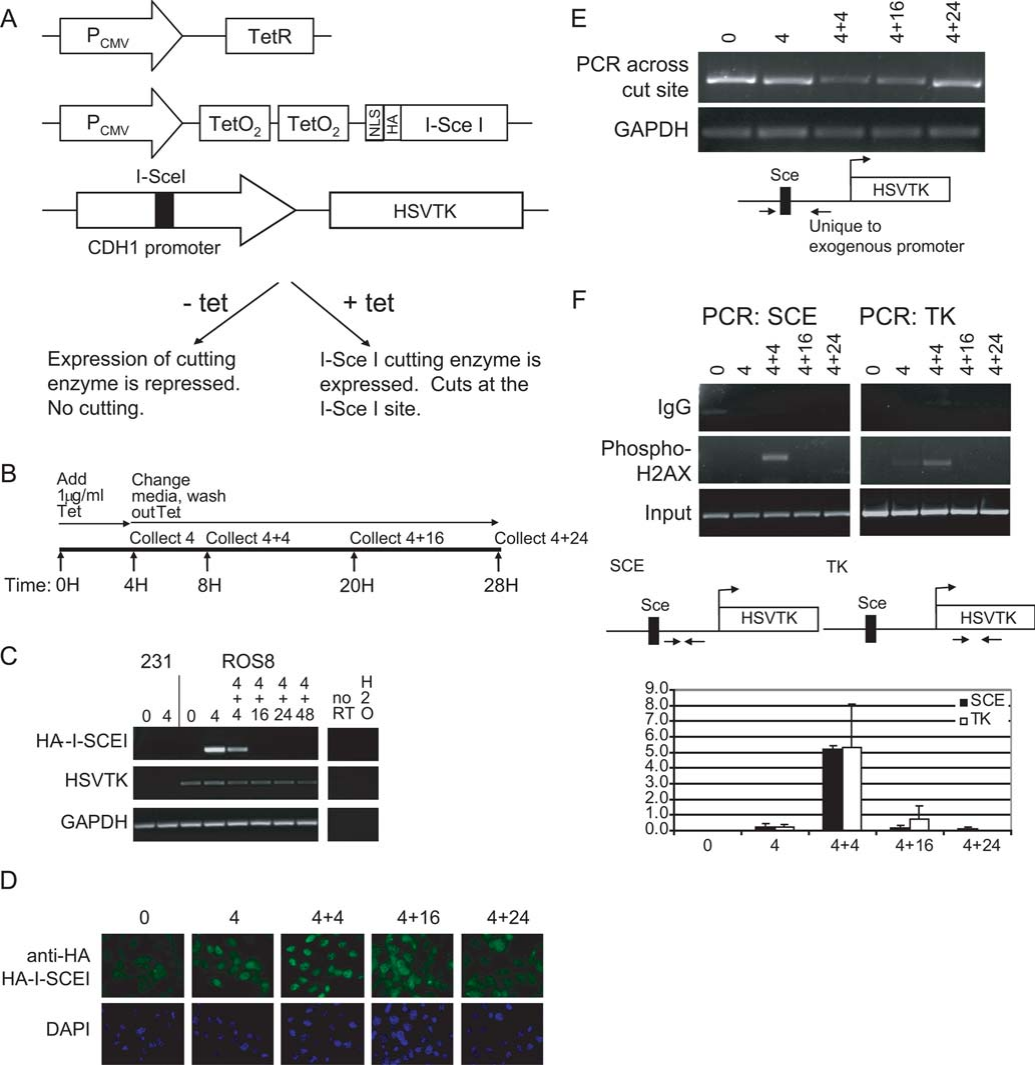
Treatment with tetracycline induces a double strand break in the inserted E-cad promoter. (A) MDA-MB-231 cells were stably transfected with constructs expressing the tet repressor, the HA-tagged I-SceI enzyme, and the E-cadherin promoter containing the I-SceI consensus cut site driving the Herpes Simplex Virus thymidine kinase gene (HSVTK). The cell line used throughout this study containing these 3 vectors is named ROS8. (B) Time course for tet treatment. (C) Treatment of cells with 1 ug/ml tet induces expression of the HA-I-SceI enzyme by RT-PCR. Expression of HSVTK by RT-PCR remains unchanged. (D) At the 4+4 hour time point, the majority of the cells express the HA-I-SceI enzyme by immunofluorescence. Cells were fixed after treatment with tet as indicated. HA-I-SceI enzyme localization was determined using an anti-HA primary antibody followed by an anti-rabbit FITC secondary antibody (green). Blue = nuclear DAPI stain. (E) DNA was collected from cells treated with tet as indicated. PCR was performed using primers on either side of the cut site with the 3′ primer being unique for the exogenous E-cad promoter. (F) Cells treated with tet were analyzed via ChIP for the enrichment of phospho-H2AX using primers in the promoter region (labeled SCE) and using primers in the gene sequence (labeled TK). The average change in phospho-H2AX recruitment over input as measured by ChIP was quantitated by gel densitometry with error bars indicating the standard error of 3 PCRs.

In order to induce a defined DSB in mammalian cells, we utilized the homing endonuclease I-SceI that has an 18 base pair recognition sequence [46]. MB-MDA-231 cells were first transfected with a tetracycline (tet) repressor plasmid and a tet operon plasmid that drives the expression of the hemagglutinin (HA)-tagged I-SceI endonuclease (Figure 1A). A single clone was selected that had no basal level of HA-I-SceI expression but had a high level of tet-induced expression. This clone was stably transfected with a plasmid that contains a consensus I-SceI cut site inserted into a copy of the E-cad promoter, containing a CpG island often DNA hypermethylated in multiple human tumor types including the MB-MDA-231 cell line [45]. The promoter drives the expression of the herpes simplex virus gene, thymidine kinase (HSVTK). A single copy clone was then tested for inducible expression of the enzyme by adding tet for 4 hours followed by washing out the tet and collecting cells at indicated intervals (Figure 1B). By RT-PCR analyses, HA-I-SceI was induced after 4 hours of tet treatment, and this expression was maintained after a 4 hour wash (Figure 1C). By immunofluorescence each cell was shown to express high levels of nuclear HA-I-SceI protein at the 4+4 hour time point (Figure 1D).

To determine the timing of the DSB formation and repair, the HA-I-SceI induced breaks were monitored by a PCR assay with primers spanning the cut site. Using this PCR, only uncut or repaired DNA will result in a PCR product. The PCR product was slightly decreased at the 4 hour time point, followed by a more substantial decrease at the 4+4 hour time point, which corresponds to the induction of the enzyme by RT-PCR (Figure 1E). The PCR product level increased at the 4+24 hour time point suggesting that a significant portion of the cells repair the DNA break during this time frame. Within minutes of damage, H2AX is phosphorylated on its C-terminal residue serine 139 at the site of DNA damage [47]. Phospho-H2AX plays a role in stabilizing repair foci containing DNA repair factors, and the mark is maintained at the break site until the break is repaired [47],[48]. Therefore, phospho-H2AX foci are a way to monitor DNA damage and repair. By chromatin immunoprecipitation (ChIP) following induction of the cutting enzyme, phospho-H2AX was localized to the DNA near the DSB at the 4+4 hour time point (Figure 1F). The presence of phospho-H2AX also suggests that not only did the break occur but that the chromatin around the break was modified as expected. Also, because both the greatest phospho-H2AX localization and the greatest amount of cut DNA as determined by PCR occur at the same time point, 4+4 hours, it suggests that this time point represents the immediate response to double strand breaks in contrast to a response to unrepaired persistent lesions that might be present at later time points [49].

**Figure 2:**
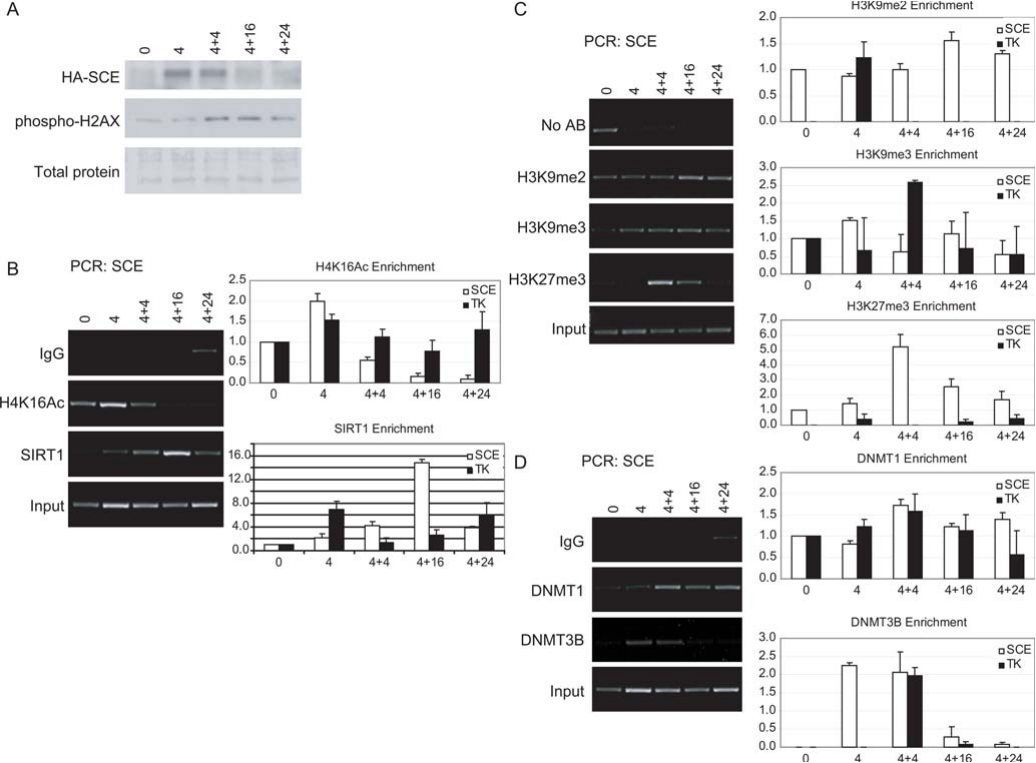
DSB damage and/or repair induces the transient recruitment of SIRT1, DNMT1, and DNMT3B. (A) Cells were treated with tet as indicated, crosslinked and sonicated for ChIP. A western blot for HA-I-SceI and phospho-H2AX was performed using a portion of the sonicated material. (B) SIRT1 is localized to the DNA in the vicinity of the cut site following DNA damage. ChIP was performed using the material from (A) and antibodies against SIRT1 and H4K16ac. Representative gels (left panel) are shown for PCR using the promoter SCE primers. Graphs (right panel) are shown, using primers for the SCE and TK regions, for the quantitative average change in recruitment over input as measured by gel densitometry. Error bars indicate the standard error for three or four PCRs. (C) Silent chromatin marks are observed transiently in the vicinity of the cut site. ChIP was performed using the material treated as in (A) and employing antibodies against di- and trimethyl H3K9 and H3K27me3. Data is presented as in (B). Error bars indicate the standard error for four PCRs. (D) DNMTs are localized to the chromatin near the cut site. ChIP was performed using the material treated as in (A) and employing antibodies against DNMT1 and DNMT3B. Data is presented as in (B). Error bars indicate the standard error for three PCRs.

As part of the ARR model of DSB repair, the restoration phase may require the recruitment of histone marks indicative of closed chromatin, as well as the proteins responsible for establishing these histone marks [14],[19],[20]. We examined the enrichment of histone marks and the recruitment of chromatin-binding proteins after inducing the DSB to determine if chromatin takes on characteristics of closed chromatin after DNA damage. Using protein from the sonicated material for ChIP, we confirmed that the HA-I-SceI enzyme was expressed at the 4 hour and 4+4 hour time point, corresponding to induction of phospho-H2AX at the 4+4 hour time point (Figure 2A). As previously introduced, SIRT1 is a stress response protein associated with DNA repair in yeast [19],[50] and transcriptional repression [39], and is a component, in *drosophila* and mammalian cells, of the PcG silencing complex PRC4 [43]. SIRT1 recruited transiently to the DNA near the break (SCE PCR) increased from the 4 hour time point to the 4+16 hour time point. The highest recruitment levels correspond to when the DNA begins to be repaired in our experimental design (Figure 2B – right lower panel). A lesser and earlier increase occurred at the downstream TK gene site which persisted to a varying degree over 24 hours (Figure 2B – right lower panel). Importantly, H4K16ac, the residue that SIRT1 is known to deacetylate, shows an early increase in enrichment at 4 hours followed by a decrease at the 4+4 hour time point, particularly at the SCE site (Figure 2B – upper right panel). The most significant decrease in the enrichment of H4K16ac corresponds to the sharp increase in SIRT1 recruitment at the 4+16 hour time point (Figure 2B – compare right upper and lower panels). We also looked for other silent chromatin marks at the break site. Importantly with respect to participation of SIRT1, we demonstrated a strong enrichment of H3K27me3, the mark catalyzed by the EZH2 enzyme in PRC4 in the absence of histone H1, again primarily to the area near the break site. There was also a less substantial enrichment of the repressive mark K9H3me2 at the SCE region at the 4+16 hour time point and in the TK region at the 4 hour time point of I-SceI induction. In addition, H3K9me3 increased sharply in the same TK region at the 4+4 hour time point (Figure 2C).

After DNA repair, in addition to changes in and restoration of histone modifications, we were particularly interested in the possible recruitment of DNMTs to the promoter after DNA damage because DNA methylation is abnormally increased at the E-cad promoter in many cancers [45]. In previous studies, using a model of UVA laser microirradiation, the DNA methylation catalyzing enzyme DNMT1 has been shown to be localized grossly to the regions irradiated immediately following damage [24]. Therefore, we looked for localization of this maintenance DNA methylation enzyme plus the de novo DNMT, 3B, to the break site. DNMT1 was localized to the break, in modest increases, mostly at the 4+4 hour time point for both the SCE and TK regions and interestingly this enrichment is maintained at the SCE site only at later time points (Figure 2D). On the other hand, DNMT3B was localized to the break site only early in the time course. Enrichment was demonstrated at the 4 hour time point only at the SCE region and at both the SCE and TK regions at the 4+4 hour time point when the I-SceI enzyme expression is the highest and the DNA is undergoing cutting.

**Figure 3:**
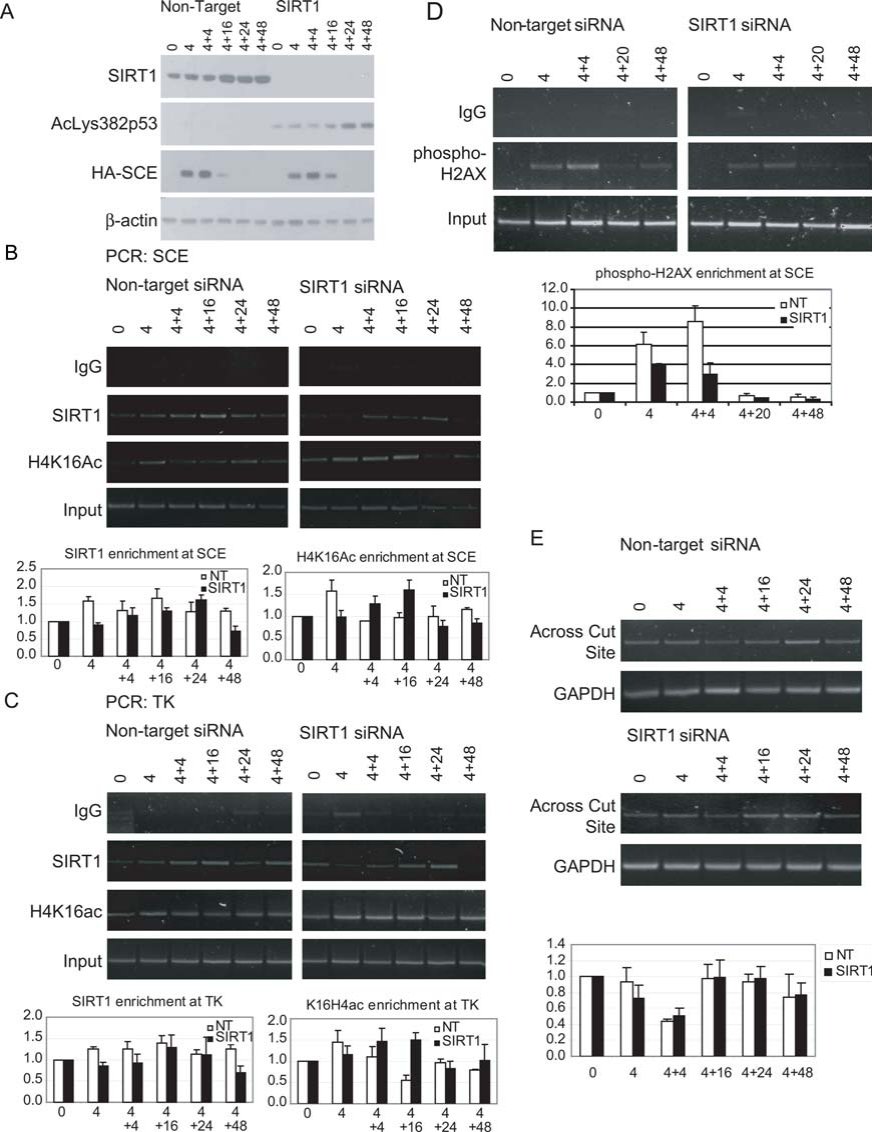
Effects of knockdown of SIRT1 by siRNA. (A) Cells were transiently transfected for three consecutive days with non-target siRNA (NT) or SIRT1 siRNA (SIRT1), treated with tetracycline to induce HA-I-SceI as indicated, and crosslinked and sonicated for ChIP analyses. A western blot was performed for anti-SIRT1, anti-acetyl lysine 382 of p53, anti-HA, and anti-β-actin using a portion of the sonicated sample. (B) Changes in ChIP results at the SCE site with SIRT1 knockdown. ChIP was performed using material from (A) and antibodies against SIRT1 and H4K16ac. Representative gels (top panels) are shown for PCR using primers in the SCE promoter region. The average change in recruitment to the promoter over input as measured by ChIP was quantitated (bottom panels) by gel densitometry for three PCRs with error bars indicating the standard error. (C) Changes in ChIP results at the TK site with SIRT1 knockdown. Using ChIP samples from (B) PCR was performed using primers in the body of the gene labeled TK with representative gels (top panels) and quantitation of results as in (B) (bottom panels). (D) Effects of knockdown of SIRT1 on phospho-H2AX recruitment kinetics. ChIP was performed using antibodies against phospho-H2AX. Representative gels (top panels) are shown for PCR using primers in the promoter region. Graphs for quantitation (bottom panel) are shown using the SCE primers, and error bars indicate the standard error for three PCRs. (E) Effects of knockdown of SIRT1 on the kinetics of break repair. Input DNA was used from the ChIP samples from (B). PCR was performed using primers on either side of the cut site with the 3′ primer being unique to the exogenous E-cad promoter. PCR using genomic GAPDH primers is used as a loading control. The average change in PCR across the break site over GAPDH was quantitated by gel densitometry for four PCRs with error bars indicating standard error (bottom panel).

We next looked to further understand the potential interactive roles of the demonstrated recruitment of SIRT1, the DNMTs, and histone modifications to the promoter in the function and DNA methylation of the promoter. We initially focused on the role of SIRT1 in the kinetics of break repair by knocking down levels of this protein. By western blot, overall levels of cellular SIRT1 were significantly knocked down in SIRT1 small interfering RNA (siRNA) treated cells versus the non-target (NT) treated cells (Figure 3A). In addition to its role in deacetylating histones SIRT1 can also deacetylate p53, and we used this latter modification to further monitor the efficacy of our knockdown. In the SIRT1 knockdown cells there is a significant increase in acetyl lysine 382 p53 consistent with SIRT1 depletion. Importantly, in MDA-MB-231 cells, p53 contains a point mutation that makes the protein non-functional, so this increase in acetyl p53 has no functional consequence [51].

In our SIRT1 knockdown studies, it is first important to note that the kinetics of SIRT1 recruitment to the DSB is somewhat different from those shown in Figure 2, possibly because the rounds of transfection necessary for the siRNA knockdown additionally stress the cells. Thus, in the non-target control (NT) cells, SIRT1 recruitment is seen at the SCE and TK sites (Figures 3B and C) earlier than in the studies in Figure 2B, peaking at 4 hours, and being maintained over 48 hours. Even though, in the SIRT1 knock down studies, there is a striking reduction of overall levels of the cellular SIRT1 protein (Figure 3A), the reduction at the SCE and TK sites, relative to that in the control NT cells, was less severe (Figure 3B and C). However, this reduction did correlate with changes in levels of H4K16ac. Thus, overall, SIRT1 localization appeared delayed at the promoter region in the SIRT1 knockdown cells as compared to the NT cells, and this correlated with sustained enrichment of the H4K16ac mark early after I-SceI induction and lasting through the 4+16 hour time point (Figure 3B). Importantly, at the 4+24 hour time point where we see late enrichment of SIRT1 at the SCE site in the knockdown cells, H4K16ac levels are again reduced, suggesting that the level of acetylation of H4K16 is dependent on SIRT1 recruitment to the break site and the SIRT1 knockdown has a functional consequence. In addition, there was a modest early decrease (4 and 4+4 hour time points) of SIRT1 at the TK site, and this correlated with increased H4K16ac at the 4+4 hour and 4+16 hour time points (Figure 3C). The most informative local result for SIRT1 knockdown appears to be the levels of H4K16ac as discussed above. The persistent high levels of H4K16ac recruitment through the 4+16 time point in the knockdown cells demonstrate the effect of the SIRT1 knockdown at the chromatin near the break site.

We performed ChIP for phospho-H2AX in knockdown cells to see if the kinetics of phospho-H2AX recruitment to, and removal from, the break site were altered (Figure 3D). In the SIRT1 knockdown cells, the levels of phospho-H2AX recruitment at the SCE site were distinctly diminished by ChIP but had the same overall time frame of recruitment and loss as in the NT cells. Importantly, for the levels of knock down of SIRT1 achieved, and with the delayed recruitment of SIRT1 to the break site, there did not appear to be a significant effect on the kinetics of repair as analyzed by our PCR assay using primers that are on either side of the cut site (Figure 3E).

**Figure 4:**
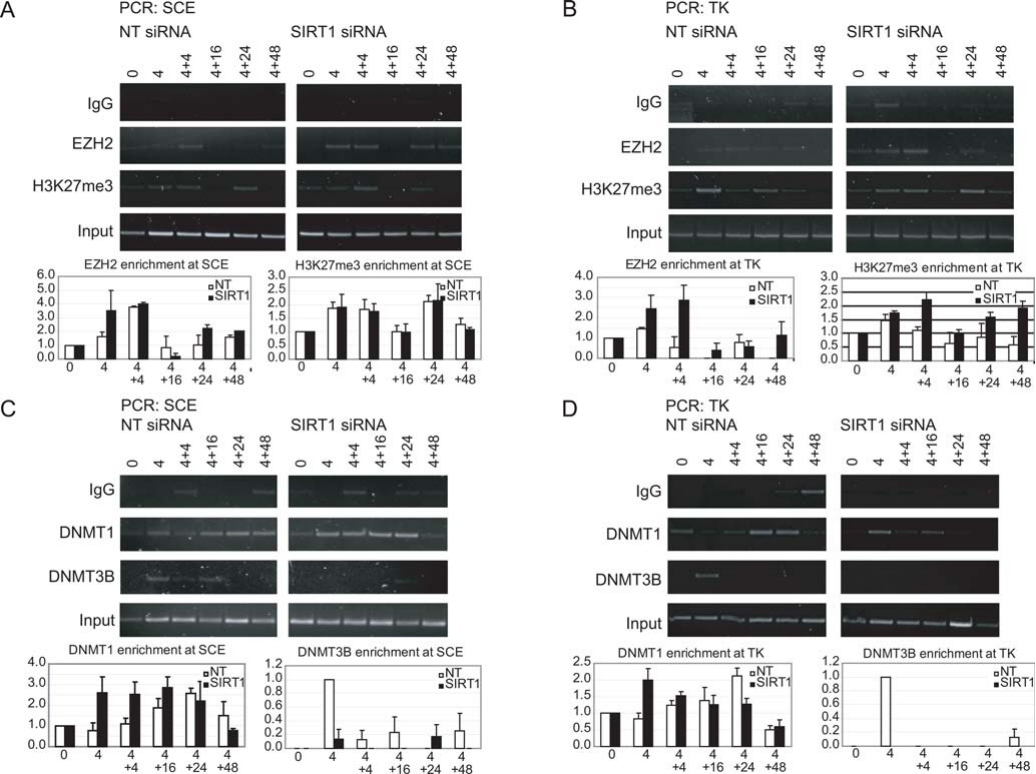
Changes in enrichment of silencing proteins and chromatin marks with knockdown of SIRT1. (A) Changes in ChIP results at the SCE site with SIRT1 knockdown. Using sonicated material from Figure 3A, ChIP was performed using antibodies against EZH2 and H3K27me3. Representative gels (top panels) are shown for PCR using primers in the SCE promoter region. The average change in recruitment to the promoter over input as measured by ChIP was quantitated (bottom panels) by gel densitometry for three PCRs with error bars indicating the standard error. (B) Changes in ChIP results at the TK site with SIRT1 knockdown. Using ChIP samples from (A), PCR was performed using primers in the body of the gene labeled TK with representative gels (top panels) and quantitation of results as in (A) (bottom panels). (C) DNMT3B localization to the cut site is lost when SIRT1 is knocked down. Cells were treated as in (A). ChIP was performed using antibodies against DNMT1 and DNMT3B. Representative gels are shown for PCR using primers in the SCE promoter region (top panels). The average change in recruitment to the promoter over input as measured by ChIP was quantitated by gel densitometry for three to four PCRs with error bars indicating the standard error (bottom panels). (D) Using ChIP samples from (C), PCR was performed using primers in the body of the gene labeled TK with representative gels (top panels) and quantitation of results as in (C) (bottom panels).

We next examined the potential role of SIRT1 in recruitment of the PcG mark H3K27me3 and the recruitment of EZH2, the enzyme responsible for catalyzing the mark [42]. In the NT cells, EZH2 was enriched in the promoter and in the body of the gene at the 4 hour time point and, to a greater extent, at the 4+4 hour time point in the promoter (Figure 4A and 4B). H3K27me3, correspondingly, was enriched in the promoter and the body of the gene at these time points, directly corresponding to the localization of EZH2. Interestingly, in the SIRT1 knockdown cells there was an increased enrichment of EZH2 in the promoter at 4 hours as compared to NT knockdown cells, but similar levels of H3K27me3 over the entire time course (Figure 4A and 4B). In contrast, in the TK gene, EZH2 enrichment was sharply increased at the 4 and 4+4 hour time points in the SIRT1 knockdown as compared to the NT cells and, correspondingly, the H3K27me3 mark was greater at all time points in the body of the gene. Thus, even modest SIRT1 knockdown appears to increase the magnitude of recruitment of EZH2 to the break region downstream to the actual cut site. Recruitment of H3K27me3 in the promoter of both the NT and SIRT1 knockdown cells decreased at 4+16 hours but increased again at 4+24 hours. This later increase may be indicative of some persistent double strand breaks. Alternatively, this increase may reflect altered kinetics observed selectively in the knockdown experiments. Both the NT and SIRT1 knockdown cells have similar H3K27me3 enrichment whereas the non-siRNA treated cells (Figure 2) show no enrichment for this modification at this later time point.

To determine whether there is any dependence, on SIRT1, of DNMT1 and DNMT3B recruitment to the DSB, we examined localization in the NT and SIRT1 siRNA treated cells. In the SIRT1 knockdown cells, there was a much increased enrichment of DNMT1 which occurred earlier than in the NT treated cells—at the 4 and 4+4 hour time point—and persisted through the 4+24 hour time point in the promoter and mostly at the 4 hour time point in the gene (Figure 4C and 4D). These data suggest that DNMT1 can be recruited, possibly to an increased degree and with slightly different timing, to the area around the break when SIRT1 levels are reduced. The most striking change, however, was that DNMT3B recruitment, even with the modest change in SIRT1 knockdown, was virtually absent in the SIRT1 knock down cells. These data suggest that SIRT1 may play a role in early recruitment of DNMT3B to the DNA around the DSB.

We next sought to further place the above findings for changes in chromatin surrounding an induced DSB into the context of genes that are DNA hypermethylated and heritably silenced in cancer—and for which our engineered E-cad promoter region provides a model. Despite the dynamic chromatin changes and DNMT recruitment we have outlined above, we saw no evidence during the acute period of DSB repair of any induction of the cancer-related gene silencing events (i.e. loss of TK expression-Figure 1C and/or DNA methylation-data not shown). However, models for how these events may take place in native cancer evolution [52],[53], and experimental models for acute, transient silencing of genes [40],[54],[55] suggest that the transient state of silent chromatin in the gene promoter region results in rare instances wherein the silenced chromatin is maintained to produce seeding of DNA methylation and permanent silencing of the downstream gene. We thus tested this hypothesis for our model.

**Figure 5:**
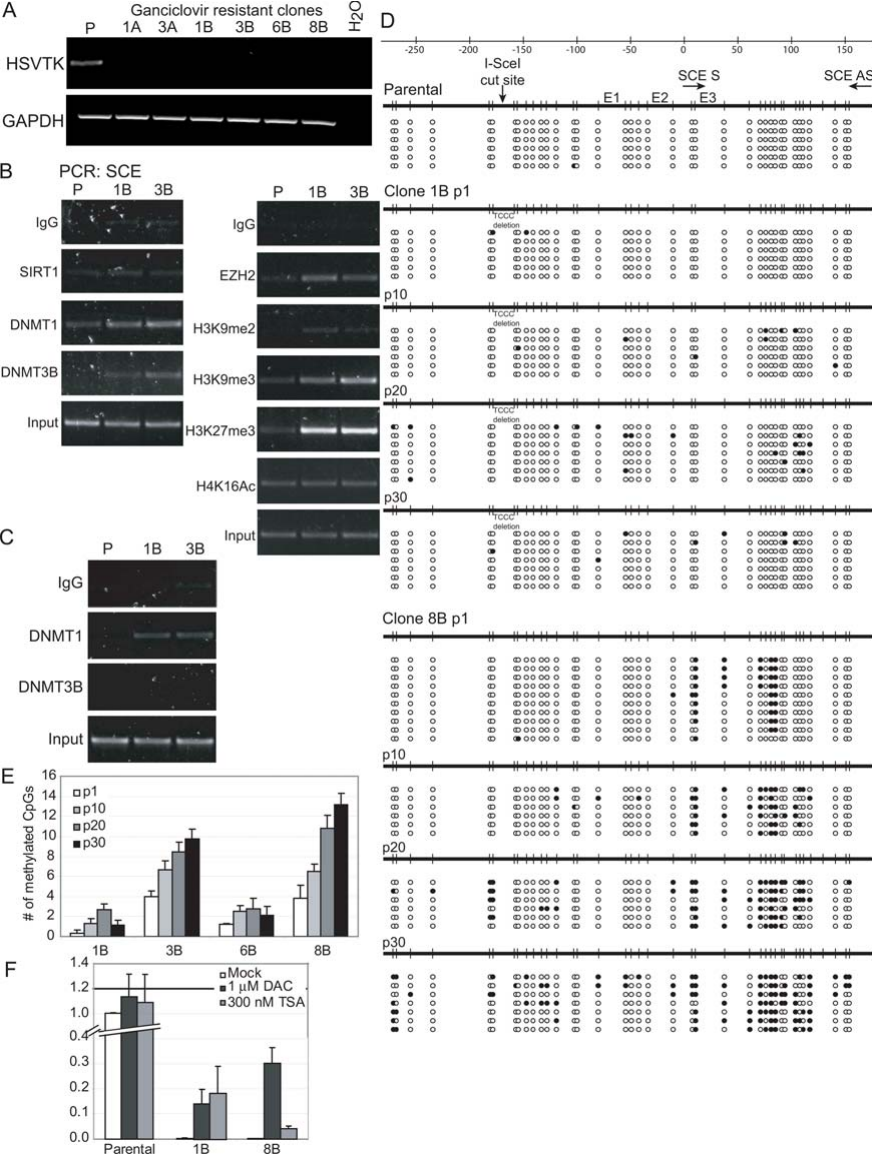
Inducing a DSB in a promoter can lead to silencing and the seeding of methylation. (A) Cells were untreated (P) or treated for 4 hours (named A) or 24 hours (named B) with tetracycline. Silencing of HSVTK was then selected for by ganciclovir treatment. Clones that survived selection were analyzed by RTPCR for HSVTK and GAPDH expression. (B) Early passages of HSVTK silent clones have an enrichment of DNMT1, DNMT3B, EZH2 and silent chromatin marks at the SCE promoter. ChIP assays for all marks in the figure were performed on cells where no DSB was induced and no cell selection was initiated (P) and two HSVTK silent clones five passages after selection with ganciclovir (1B and 3B). Representative gels are shown for PCR using primers in the SCE promoter region. (C) Late passages (p34 to p36 after selection with ganciclovir) of HSVTK silent clones show enrichment for DNMT1 but not DNMT3B. ChIP was performed with cells as in (B). Representative gels are shown for PCR using primers in the SCE promoter region. (D) Bisulfite sequencing data for DNA methylation status of clones. DNA was isolated from uncut, unselected cells (parental) and two HSVTK silent clones (1B and 8B) at passages 1, 10, 20 and 30 after ganciclovir selection. Bisulfite sequencing was performed using primers on either side of the cut site with the 3′ primer being specific for the exogenous E-cad promoter. Open circles indicate unmethylated CpGs and closed circles indicate methylated CpGs. The location of the Sce cut site, the transcription start site, the SCE primers used for ChIP, and the well-characterized E-cad E-boxes (E1, E2, and E3) are indicated. (E) CpG methylation spreads with passage. The E-cad promoters containing the cut site were bisulfite sequenced in HSVTK silent clones at passages 1, 10, 20 and 30 after ganciclovir selection. A mean number of methylated CpGs per bisulfite sequenced clone is reported. A minimum of 6 bisulfite clones were sequenced per HSVTK silenced clone. The means presented are determined from the data shown in (D) plus additional unmethylated clone 6B and methylated clone 3B. (F) Effects of DAC and TSA treatment on expression of HSVTK as analyzed by realtime RT-PCR. In the lesser DNA methylated clone, 1B, both drugs lead to increased TK expression, while in the more DNA methylated clone, 8B (see panels D and E), DAC induces more increased expression than TSA treatment. Parent clones, or passage 30 of HSVTK silent clones, were treated with 1 µM deoxyazacytidine once a day for three days or once with 300 nM TSA for 16 hours. Realtime RT-PCR was performed for HSVTK expression. The mean HSVTK expression is shown in relation to expression in untreated parental cells with error bars indicating the standard error for three independent experiments.

To look for selection of long term silencing events, we induced our DSB by treating the cells with tet for either 4 hours or 24 hours and then negatively selected the cells for HSVTK silencing via treatment with ganciclovir. Cells that silence the E-cad promoter do not express HSVTK and therefore are not sensitive to ganciclovir, unlike the parental, uncut cell line. After selecting one thousand cells that were either uncut or cut with ganciclovir, no clones from the uncut cells survived whereas ten clones from the 1000 cells plated from the cut cells survived. As an additional control experiment, a cell line containing the inducible I-SceI enzyme and an E-cad promoter without an I-SceI consensus cut site driving the expression of the HSVTK gene was treated with tetracycline followed by ganciclovir as above. No clones survived from this cell line (data not shown). By RTPCR expression levels, HSVTK was transcriptionally silent in the ganciclovir resistant clones from above (Figure 5A). Interestingly, one out of the ten silent clones had a portion of the promoter or gene deleted (data not shown), indicating that improper repair may result in deletion events. The rest of the silent clones appeared to have intact sequences as examined by PCR. Therefore, the frequency of silencing without deletion of HSVTK in our system is 0.9%. We examined the promoters in two of these silent clones by ChIP at passage 5 after ganciclovir selection. There was no enrichment of SIRT1 or change in H4K16ac in the clones as compared to uncut, unselected cells (P), but there was a significant enrichment of DNMT1, DNMT3B and EZH2, along with the silent chromatin marks dimethyl and trimethyl H3K9 and H3K27me3 (Figure 5B). Interestingly, when the promoters were examined by ChIP at a later passage (p34 to p36 after ganciclovir selection) DNMT1 was still enriched at the promoter, but localization of DNMT3B was lost (Figure 5C). These findings suggest that the chromatin in the promoter is indeed in a silent state and that this silent state is accompanied in later passages by the presence DNMT1 but not DNMT3B.

**Figure 6:**
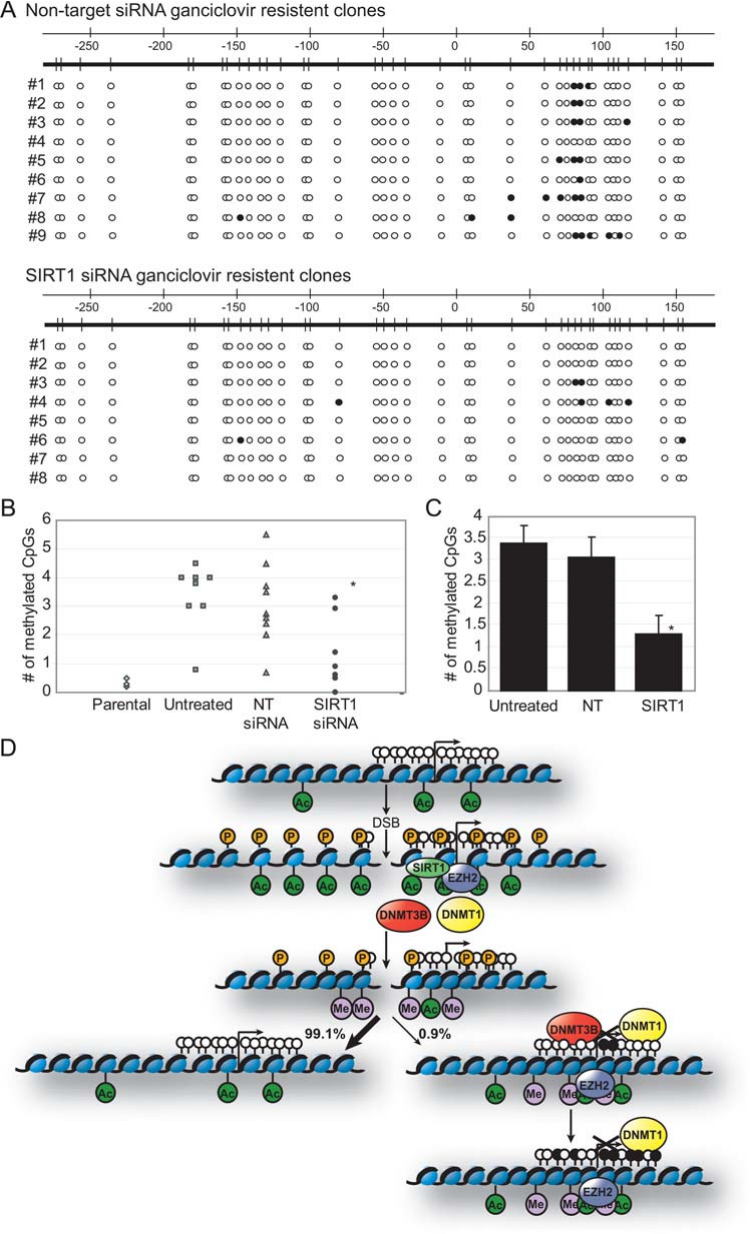
Reduction of SIRT1 during DNA damage decreases the number of silent clones that have methylation. (A) Cells were treated for three consecutive days with non-target (NT) or SIRT1 siRNA followed by 24 hours of tetracycline. Cells were then selected for silencing of the HSVTK gene by treatment with ganciclovir. DNA was isolated from clones that survived ganciclovir treatment. Bisulfite sequencing was performed as outlined in Figure 5. One representative bisulfite sequenced clone is presented for each HSVTK silent clone. (B) A dot plot of the mean number of CpGs methylated per HSVTK silenced clone from either un-siRNA treated cells (untreated) (Figure 5D & E), non-target siRNA treated cells (NT), or SIRT1 siRNA treated cells (SIRT1). The number of clones without methylation versus the number with methylation is significantly different between the SIRT1 knockout cells and the non-target cells (the asterisk indicates p<.05 by chi-square test). (C) The mean number of methylated CpGs per all HSVTK silent clones from either un-siRNA treated cells, NT siRNA treated cells, or SIRT1 siRNA treated cells. Error bars indicate the standard error. The difference in the mean for the SIRT1 siRNA treated cells is significantly different from that of the NT siRNA treated cells (the asterisk indicates p<.05 by Student's T-test). (D) Model for double strand break induced silencing of a gene. Initially after a DSB occurs in the promoter of a gene H2AX is phosphorylated (orange circles) and H4K16 is acetylated (green circles) causing the chromatin to open, allowing access to the break by repair factors and a stimulation of DNA damage signaling. Then SIRT1, EZH2, DNMT1, and DNMT3B are recruited to the area around the break site resulting in a decrease in H4K16ac and an increase in H3K27me3 (purple circles). These modifications result in compaction of the chromatin around the break site possibly causing a reduction in DNA damage signaling initiated by the prior decondensation of the chromatin or preventing transcription of unrepaired DNA. In the majority of cells (99.1%) the DNA is repaired and the chromatin returns to its original state. In a small fraction of the cells (0.9%) the promoter becomes silenced and gene expression is lost possibly due to the persistent localization of EZH2, DNMT1, and DNMT3B to the area of the break site and the prolonged condensed chromatin. Additionally, there is a seeding of DNA methylation in the area 3′ to the break site (white circles – unmethylated CpGs; black circles – methylated CpGs). After passage, DNMT3B is no longer localized to the promoter but EZH2 and DNMT1 are retained. The DNA methylation continues to spread further stabilizing the silencing of the downstream gene.

Using a PCR that was specific for the I-SceI containing E-cad promoter, we bisulfite sequenced the promoter to examine the DNA methylation status of the HSVTK silent clones. The parental cell line was almost completely unmethylated (Figure 5D). The HSVTK silent clones showed a varying degree of methylation. HSVTK silent clones originally treated with tet for 4 hours showed very little CpG methylation (data not shown), however, the majority of those treated with tet for 24 hours showed an increase in CpG methylation 3′ to the break site (Figure 5D and 6B). To examine how methylation might change with time in the silenced clones, we bisulfite sequenced increasing passages of two HSVTK silent clones, one without and one with initial DNA methylation (Figure 5D). Clone 1B, which initially had very little methylation continued to have only a scattered change in methylation with passage. Clone 8B had initial methylation just 3′ to the break site and methylation spread with passage towards the actual break site and became quite prominent by passage 30 (Figure 5D). Interestingly, this methylation occurs in the region that is flanked by the ChIP PCR primers in the promoter (SCE), demonstrating that the DNA methylation enzymes are recruited to the region where the methylation is occurring (Figure 5D). To further demonstrate how the DNA methylation changes with cell passage, we calculated the mean number of methylated CpGs per bisulfite-sequenced clone per passage of selected HSVTK silent clones (Figure 5E). Clones with little initial methylation (clones 1B and 6B) showed almost no increase in the mean number of methylated CpGs per bisulfite sequenced clone (Figure 5E). However the HSVTK silent clones with an initial methylation of 3–4 CpGs (clones 3B and 8B) gained methylation with increasing passage (Figure 5E).

To further look at the nature of the relationships between silencing and DNA methylation, we treated one unmethylated and one DNA methylated clone with the DNA demethylation agent 5-deoxy-azacytidine (DAC) or with Trichostatin A (TSA), a type I/II histone deacetylase inhibitor. DAC treatment inhibits DNMT activity and causes re-expression of genes silenced with DNA methylation [56],[57]. It will sometimes cause this response in low expression genes which have no proximal promoter DNA methylation [57]. However, we and others have previously shown that TSA treatment is generally ineffective for re-expression of such silent genes, particularly when the CpG island is densely DNA methylated [56]. TSA can be more effective when the DNA methylation in such genes is partial or minimal [56]. Interestingly, when the HSVTK silent clones are treated with TSA or DAC, the clone that has silent chromatin but no methylation (clone 1B) has re-expression of HSVTK by either treatment (Figure 5F). However, clone 8B, which has silent chromatin and increased DNA methylation, has HSVTK re-expressed by DAC treatment but to a much lesser degree with TSA. These findings suggest that at this later passage of clone 8B the partial DNA methylation plays at least some role in maintaining the silencing of the HSVTK gene.

As demonstrated above, a DSB in the promoter of a gene that is associated with transient recruitment of silencing proteins can, in occasional cells, cause long term silencing of the involved gene. Some partial DNA methylation can also be associated with such silencing and is possibly maintained by the persistence of the maintenance DNMT, DNMT1, in the region. Because we observed in the acute DSB induction studies a transient recruitment of the de novo DNMT, 3B—which would be the best candidate to initiate any DNA methylation—and evidence that SIRT1 may play a role in this recruitment, we sought to determine the significance of these dynamics in long term silencing. To study this, we performed our siRNA knock down of SIRT1 by treating cells with NT or SIRT1 siRNA, followed by treatment with tet for 24 hours, then selection with ganciclovir. Global SIRT1 knockdown levels were similar after tet treatment to those in the studies described earlier (Figure 3A). Both NT and SIRT1 siRNA treated cells had similar numbers of surviving, silenced clones (9 and 8 out of approximately 1000 cells selected, respectively) suggesting that the amount of reduction achieved for SIRT1 recruited to the promoter during DNA damage did not alter silencing of the promoter. Next, the DNA from clones that survived ganciclovir treatment was bisulfite treated and sequenced as above. NT treated HSVTK silent clones showed a similar pattern to non-siRNA treated cells (untreated) both in terms of how many bases were DNA methylated per clone and the position where the methylation occurred (Figure 6A and 6B). Thus, CpGs 3′ to the cut site were methylated in 8 out of 9 of the clones with a mean of 3.1 methylated CpGs per clone (Figure 6A and 6C). In HSVTK silent clones from SIRT1 siRNA treated cells, only 2 out of 8 clones had methylated CpGs in numbers greater than those for uncut cells. This difference in the number of HSVTK silent clones with methylated CpGs versus those with unmethylated CpGs was significantly different between the SIRT1 knockdown cells and either the NT or the untreated cells (Figure 6B). Also, the mean number of methylated CpGs per HSVTK silent clone in SIRT1 knockdown cells, 1.3, is significantly different from the number in both the NT knockdown cells, 3.1 methylated CpGs, and the cells not treated with siRNA, 3.4 methylated CpGs with p<.05 by Student's T-test (Figure 6C). The above results suggest that the reduced levels of SIRT1 recruited to the break site during repair do not affect silencing of the promoter. However, possibly by playing a role in transient recruitment of DNMT3B to the DSB region, SIRT1 does appear to play a role in seeding of methylation in the promoter CpG island in occasional cells, which can then be perpetuated and expanded by the persistent presence of DNMT1.

## Discussion

In the present study our data emphasize, as has been shown for some chromatin constituents by others [19],[24],[29],[30], that during normal repair of a DSB, silencing proteins are recruited to the site of DNA damage along with enrichment of their corresponding histone marks. We substantially add to these previous data by showing the involvement of the principal long term silencing complex PcG. In the ARR model of DSB repair SIRT1 and the PcG protein, EZH2, most likely play a role in the restoration phase of repair by returning chromatin back to its original more condensed state or making chromatin even more condensed (Figure 6D). We hypothesize that following DNA damage in our particular model involving a gene promoter region, the EZH2 catalyzed trimethylation of H3K27, plus the enrichment of the additional silencing marks, H3K9me2 and H3K9me3, may all lead to a transient silencing of the gene in order to make sure the DNA repair is complete before transcription can resume and/or to a compaction of the chromatin that blunts the DNA damage signaling stimulated by the initial opening of the chromatin [23].

During the normal process of DSB repair the association of the above proteins and histone marks with the DSB appears to be transient for most cells, returning to low or absent baseline levels after repair has occurred. This is true in our exogenous gene promoter region, even in a tumor cell which involves a promoter sequence that frequently is DNA hypermethylated and abnormally, heritably silenced in cancer. However, and important to the model for how abnormal CpG island DNA hypermethylation and gene silencing might occur in cancer, we have demonstrated that induction of a break in the promoter of a gene can infrequently lead to long term silencing of that gene. Silencing could occur because, occasionally, there is permanent association of the silencing factors to the break or at least proteins that are important for establishing an epigenetic memory for silencing. Additionally, in cells with such retained silenced promoters, there appears to be an early seeding of CpG methylation that spreads over time and which potentially can, then, contribute to a more stable silencing of the promoter.

This work suggests that a DSB occurring in the promoter of a gene may be an initiating event for the silencing of the promoter, leading to a mechanism by which oxidative or other DNA damage can induce epigenetic silencing, including promoter CpG island DNA hypermethylation of tumor suppressor genes. In this regard, SIRT1 is a key stress response and cell survival protein [35],[38]. This protein has now been associated in stem/precursor and cancer cells with silencing chromatin [40] including PcG complexes [43],[58], with DNA damage repair in multiple settings [59],[60], and, in our own studies, with maintenance of gene silencing for DNA hypermethylated cancer genes [39]. We [61] and others [62],[63] have recently reported that there is an association of embryonic stem cell like repressive chromatin patterns for large groups of such cancer genes, and particularly PcG components and the corresponding histone modification, H3K27me3. Furthermore, we have hypothesized from studies of cancer progression, including the very early appearance of many DNA hypermethylated genes, that this PcG component is particularly important for the vulnerability of genes to the abnormal DNA methylation during cancer evolution [64]. In turn, we have wondered whether settings that are high risk for cancer development, such as chronic inflammation which exposes cells to a significant amount of DNA damage, collaborate with the PcG chromatin for such DNA methylation recruitment. Our present study further points to this possibility and links SIRT1 to the process, especially to recruitment of DNA methylation. The findings suggest that a DSB occurring in the promoter of a gene may initiate epigenetic silencing in occasional cells and this silencing, in turn, could contribute risk of tumor development.

Our present link of SIRT1 and PcG to the DNMTs during DNA damage repair brings up important issues regarding whether these proteins form a complex during DNA repair or are recruited independently to the break site. A PcG complex, termed PRC4, containing SIRT1 and EZH2, has previously been identified [43]. Additionally, SIRT1 has been found to co-localize and to be co-immunoprecipitated with DNMT1 at rRNA [65]and it has been hypothesized that the DNMTs may be recruited to DNA through interaction with PcG [62],[66]. Although SIRT1 and EZH2 appear to be recruited to the break site in the same time frame, EZH2 is still recruited, and possibly even more so, when SIRT1 is knocked down, suggesting that its recruitment is not dependent on SIRT1. There are higher levels of EZH2 enrichment in the TK gene in the SIRT1 knockdown cells in contrast to relatively low EZH2 enrichment in the TK gene in the non-target knock down cells. The presence of higher levels of EZH2 and H3K27me3 may be an attempt to further compact the DNA in the absence of high levels of SIRT1 or to turn off the DNA damage signal which may have been initiated by histone acetylation and therefore maintained in the SIRT1 knockdown cells. Intriguingly, an important and novel finding in our studies is that the seeding of the DNA methylation appears highly dependent on SIRT1 presence during the acute DNA damage and repair interval and seems likely to involve a role for SIRT1 in the transient localization of the de novo DNMT, 3B, during repair. It is unclear from this work whether this recruitment of DNMT3B is because of a direct interaction with SIRT1. After DNA damage, the earliest time points of SIRT1 recruitment (4 and 4+4 hours-Figure 2B) correspond to the time points where we demonstrate enrichment of DNMT3B (Figure 2D). However, DNMT3B is not enriched at later time points where SIRT1 recruitment is the greatest. As an alternative to a direct physical interaction between SIRT1 and DNMT3B, SIRT1 could affect DNMT3B localization to the cut site indirectly by playing a critical role in a complex that forms at the break site or by modifying another protein that plays a role in DNMT3B recruitment. For example, SIRT1 knockdown appears to have the greatest effect on the persistence of H4K16ac after damage. Acetyl H4K16 is a critical residue for chromatin formation, with deactylation of this residue being associated with tight compaction of chromatin [67]. Its status could influence other histone modifications through composition of the complex formed at the break site that could potentially contain HATs, HDACs, HMTs, and/or histone demethylases. A change in these other histone modifications could in turn influence DNMT3B recruitment. Unlike DNMT3B, DNMT1 localization is independent of SIRT1, suggesting that DNMT1 is recruited through a different mechanism. Further studies need to be performed to identify how silencing complexes formed at sites of DNA damage precisely involve interactions between SIRT1, EZH2 and other PcG components, and the DNMTs and whether such interactions are operative in other transcriptional silencing processes.

Recently Cuozzo et al also demonstrated that DNMT1 is associated with chromatin, after DNA damage, specifically after repair by HR [68]. After HR, some DNA methylation occurs that plays a role in silencing the recombined genes. This silencing is dependent on DNMT1 and reversed by treatment with DAC. Interestingly, this paper showed induced methylation localized 200 to 300 bp 3′ of the break site, similar to the degree and relative localization of DNA methylation in our model cut site [68]. We extend these findings by showing that with passage this methylation can be expanded, increasing from approximately 4 methylated CpGs to 10–13 methylated CpGs in 30 passages. We suggest that our current findings provide molecular support for our previous model [64] concerning how this expansion occurs in tumors. An event occurs in a cell that causes an initial seeding of methylation at a promoter, and over time during tumor progression, this methylation spreads and contributes to progressive stable silencing of the involved promoter. A similar model exists for transient “hit and run” silencing of a promoter construct by a transcription repression complex leading to cell clones with retained silencing, even in the absence of the original complex, and progressive spread of DNA methylation [54].

Another intriguing finding from our work is the different potential roles of DNMT1 and DNMT3B in silencing. Both are found to be transiently recruited to the site of DNA damage, albeit with slightly different timing. In terms of normal DNA repair, this transient recruitment of DNMT1 and DNMT3B to the break site may be part of a universal mechanism used during DNA damage repair to restore the correct DNA methylation code to the area around the break. However, this may be more for areas widely flanking the break in our model situation since the CpG island of gene promoters like the one we are using are generally maintained free of DNA methylation in normal cells. Alternatively, in the transient setting, these proteins could perform a silencing role without using their DNA methylating capacity since multiple studies suggest these proteins have transcriptional repression potential independent of their ability to catalyze DNA methylation [69]–[71]. Although DNMT1 is predominantly a maintenance DNMT, it has been demonstrated to have some de novo methyltransferase activity [72],[73], while DNMT3A and DNMT3B are thought to be the predominate de novo methyltransferases [74]. Our present work supports the thought that these enzymes can work together at different phases of methylation initiation, maintenance, and spreading. We only detect seeding of methylation when DNMT3B is present at the cut site and do not observe seeding when only DNMT1 is present. Additionally, at early passages of the clones that have silenced the promoter containing the cut site, both DNMT1 and DNMT3B are present. However, at later passages, enrichment of DNMT3B is lost even though the methylation is still expanding. We were not able to detect enrichment of DNMT3A either transiently or in our silent clones. It is unclear however whether these results are due to a lack of recruitment or a sensitivity issue for the antibody used for ChIP. These findings suggest that DNMT3B is important for the initial seeding of methylation, while DNMT1 is needed for de novo activity in expanding and maintaining the sites of DNA methylation.

With respect to DNA repair, chromatin modifications are important in the specific steps of repair of DSBs. Phospho-H2AX is required for the recruitment of the chromatin remodeling complex INO80 that most likely plays a role in repositioning nucleosomes around the break [75]–[77]. Phospho-H2AX is also necessary for the stable, concentrated recruitment of DNA repair proteins to the site of the break [48],[78]. In addition to H2AX modifications, it has been demonstrated by using a pan-acetyl lysine H4 antibody that lysine residues of histone H4 are acetylated by the human TIP60 histone acetyltransferase complex in response to DNA damage [33]. Our work supports these findings because at the 4 hour time point we see an increase in H4K16ac when compared to uncut cells. Adding to this process, we show that this initial acetylation of histone H4, specifically at lysine 16, is followed by the deacetylation of the same residue concomitant with recruitment of SIRT1 to the break site. We hypothesize that this deacetylation is important to return the chromatin back to its original state following DNA repair. Although we did not see a change in DNA repair following knockdown of SIRT1 and prolonged acetylation of H4K16, it has been demonstrated in yeast that a lack of deacetylation of H4K16 after DNA damage affects repair by the NHEJ pathway [79].

In mammalian cells it is hard to separate a role for SIRT1 in DNA damage repair from its role in p53 regulation. SIRT1 deacetylates p53 and therefore decreases transcriptional activity of the protein. Inhibition of SIRT1 has been shown to induce apoptosis and enhance radiation sensitization, most likely because p53 acetylation is increased [80]. Loss of SIRT1 has also been shown to allow cells to bypass senescence and allow cell division without repair of DNA [81]. While it is unclear in these previous studies how much of the effect of SIRT1 on damage sensitization and cell cycle check points is dependent on p53, our system looks at the role of this protein in DNA damage repair independent of p53. In MDA-MB-231 cells p53 is mutated so, although SIRT1 knock down causes an increase in acetyl p53, the p53 protein is non-functional [51]. Our work then directly demonstrates that SIRT1, in the absence of functional p53, is localized to the chromatin near a DSB and plays a role in recruiting DNMT3B to the vicinity.

In summary, the system of Jasin et al [46] used here uniquely allows us to determine if induction of a DSB in a promoter can lead to transcriptional silencing. In a transient setting, several factors that play a role in gene silencing are recruited to the break and, occasionally, retention of some of these factors can lead to sustained silencing, which can be associated with initiation and spreading of DNA methylation to further stabilize the silencing. Our model is important to understanding how DNA damage occurring at gene promoter sites may be one key factor in initiating abnormal epigenetic gene silencing in association with abnormal CpG island DNA methylation. In terms of cancer prevention, targeting the series of events suggested by our model at sites of chronic inflammation may be beneficial to reducing tumor formation by decreasing the silencing of the large number genes which we now know become aberrantly silenced during neoplastic progression [45],[52],[64]. Our findings suggest new molecular events to consider for cancer prevention targeting and the need for a further understanding of the complex that initiates DNA silencing and how it is recruited to promoters.

## Materials and Methods

### Cell Culture and Creation of Stable Cell Lines

MDA-MB-231 cells (ATCC, www.atcc.org) were cultured in Dulbecco's modified Eagle's medium supplemented with 10% tetracycline-tested fetal bovine serum (Hyclone, www.hyclone.com). The homing endonuclease I-SceI, along with the NLS and HA epitope tag, was amplified from the pCMV-ISceI vector [46] (a gift from M. Jasin) and inserted into the pcDNA4-TO vector (Invitrogen, www.invitrogen.com). The pEGFP1-E-cad vector contains genomic DNA corresponding to the human E-cad promoter inserted into the EcoRI/SalI sites of the pEGFP-1 vector. The consensus I-SceI cut-site was inserted into the E-cad promoter at the unique MluI restriction site that is located at −171 in relation to the transcription start site. This insertion avoids all characterized Ebox and Sp1 elements within the E-cad promoter. The EGFP coding sequence was removed and replaced with HSV thymidine kinase sequence that was amplified from the BaculoDirect N-terminal linear DNA Gateway Cassette (Invitrogen). MDA-MB-231 cells were co-transfected with pcDNA6-TR (Invitrogen) and pcDNA4-TO-HA-I-SceI and dual integration was selected for using Zeocin and Blasticidin treatment. Stable clones were isolated and screened for tetracycline induced expression of HA-I-SceI and no background expression without tetracycline. The clone with highest inducible expression was then transfected with the pCDH1-I-SceI-HSVTK vector. Clones with stable integration were selected for with G418 treatment. Clones were screened by two PCRs with linear amplification for single copy insertion of the entire sequence. To verify the copy number in the final clone selected (ROS8) we prepared copy standards using a known amount of pCDH1-I-SceI-HSVTK plasmid DNA combined with 50 µg genomic DNA from non-transgenic MDA-MB-231 cells or pGAPDH plasmid DNA only. By realtime PCR for a primer set in the TK gene or in the GAPDH gene we used the copy standards to develop two standard curves. The TK copy number in 50 µg genomic DNA from the pCDH1-I-SceI-HSVTK containing clone was normalized so the GAPDH copy number was 2 (Figure S1) [82].

### Tetracyline Treatment

For tet-induced expression of the HA-I-SceI enzyme, tet (Sigma, www.sigmaaldrich.com) was added to the culture media to a final concentration of 1 µg/ml. After 4 hours the media was removed and the cells were washed twice with PBS. Then cells were either collected (4 hour time point) or fresh media was added and the cells were incubated at 37°C for an additional 4 hours (4+4 hour time point), 16 hours (4+16 hour time point), 24 hours (4+24 hour time point), or 48 hours (4+48 hour time point). For the generation of silent clones, cells were treated with tet for 4 hours or 24 hours followed by being sub-cultured at a density of 1000 cells per 100 mm dish (cell numbers were determined by counting with a hemocytometer). Ganciclovir (Sigma) was added to the dish at a final concentration of 50 µM. Media was changed bi-weekly until single clones were observed. Silent clones were continually grown in the presence of ganciclovir. Clones originating from cells treated with 4 hours of tet were labeled A and those originating from cells treated with 24 hours tet were labeled B.

### RNA Preparation and Analysis

Total RNA was extracted (Qiagen, www.qiagen.com) according to the manufacturer's instructions and subjected to reverse transcription using Superscript II RNAse H Reverse Transcriptase (Invitrogen) followed by semi-quantitative polymerase chain reaction or quantitative real-time polymerase chain reaction. For real-time analyses, the QuantiTect SYBR Green PCR kit (Qiagen) and a BioRad iCycler (Biorad, www.bio-rad.com) were used. Values reported were based on a standard curve generated by serial dilution of the untreated parental sample, and expression was reported as a fraction of the expression in the untreated parental samples. The sequences of the primers used are listed in Table S1.

### Western Blot Preparation and Analysis

Part of the sonicated samples collected for ChIP was used for western blot. Protein concentrations were measured by BCA (Pierce Biotechnology, www.piercenet.com). Protein extracts were subjected to polyacrylamide gel electrophoresis using the 4%–12% NuPAGE gel system (Invitrogen), transferred to PVDF (Millipore, www.millipore.com) membranes, and immunoblotted using antibodies that specifically recognize SIRT1 (DB083, Delta Biolabs, www.deltabiolabs.com), HA (sc-805, Santa Cruz Biotechnology, www.scbt.com, Figure 2), HA-HRP (12013819001, Roche Applied Science, www.roche-applied-science.com, Figure 3), phospho-H2AX (05-636, Millipore Corporation), and acetylated lysine 382 p53 (Cell Signaling Technology, www.cellsignal.com).

### ChIP

ChIP analysis was performed as described previously [83] with a few modifications. Culture medium was removed, the cells were washed once with PBS, and then an additional 10 ml of PBS was added to the plate. Proteins were cross-linked to proteins by addition of disuccinimidyl glutarate (DSG, Pierce) to the PBS to a final concentration of 0.5 mM for 30 min at room temperature. Proteins were then cross-linked to DNA by addition of formaldehyde to a final concentration of 1% for 10 min at room temperature. Antibodies to SIRT1 (05-707, Figure 2B & 5B), phospho-H2AX (07-164), and H4K16ac (07-329) were obtained from Millipore. Antibodies to SIRT1 were also obtained from Delta Biolabs (DB083, Figure 3B & C). Antibodies to EZH2 were from Cell Signaling Technologies (4905). Antibodies to DNMT1 (IMG-261A) and DNMT3B (IMG-184A) were from Imgenex (www.imgenex.com). Antibodies to H3K9me2, H3K9me3, and H3K27me3 were generous gifts from T. Jenuwein. Immune complexes were collected with 100 µl of 3∶1 Protein A and Protein G magnetic Dynabeads (Invitrogen) for 2 hours at 4°C. Primers were used to amplify the promoter region of the inserted E-cad promoter (SCE). The sense primer was specific for the E-cad promoter and the anti-sense primer was specific for the E-cad promoter containing the cut site. A different set of primers was used to amplify a region in the HSVTK gene (TK). Sequences of the primers are listed in Table S1. Ten microliters of PCR product were size fractionated by PAGE and were quantified using Kodak Digital Science 1D Image Analysis software. Enrichment was calculated by taking the ratio between the net intensity of the gene promoter PCR products from each primer set for the bound, immunoprecipitated sample and the net intensity of the PCR product for the non-immunoprecipitated input sample. Values for enrichment were calculated as the average from at least three independent PCR analyses. Each ChIP experiment was performed twice. The data presented is from one representative experiment.

### SIRT1 Knockdown

Cells were transiently transfected with 25 nM non-target siRNA (D-001210-05, Dharmacon, www.dharmacon.com) or SIRT1 siRNA (L-003540-00, Dharmacon) using lipofectamine 2000 (Invitrogen) for three consecutive days following the manufacturer's suggested protocol. On the fourth day, the tet treatment schedule was started either for collection of samples at different time points or tet treatment prior to ganciclovir selection.

### 5-Aza-dC and TSA Treatments

Cells were treated with mock, 1 µM 5-Aza-dC (Sigma) for 72 hours, or with 300 nM TSA (Wako, www.wakousa.com) for 16 hours, as described previously [56].

### Bisulfite Sequencing

Bisulfite sequencing was performed as previously described [84] on DNA from parental uncut cells or clones that were ganciclovir resistant and had silenced HSVTK. Primers that are specific for bisulfite treated DNA and are methylation non-specific were used (Table S1). The sense primer is specific for the E-cad promoter. The anti-sense primer is specific for the E-cad promoter containing the cut site.

## Supporting Information

Figure S1 Clone ROS8 contains one inserted copy of pCDH1-SCE-HSVTK. (A) Gel based PCR was performed on genomic DNA collected from the parental MDA-MB-231 cell line, three different passages of the clone ROS8 (p9, p30 and p50), and serial dilutions of copy number standards using a known amount of pCDH1-I-SceI-HSVTK or GAPDH plasmid DNA. Genomic DNA primers specific for the HSVTK gene (TK), the SCE containing CDH1 promoter (SCE), or GAPDH were used. (B) Realtime PCR was performed on DNA used in (A). Two standard curves were generated using the copy number standards, one for the TK and one for the GAPDH primers from (A). The TK copy number in the genomic DNA was normalized so the GAPDH copy number was two. The means plotted are the calculated TK copy number from three independent real time experiments with error bars indicating standard error.
(1.16 MB EPS)

Table S1 Forward and reverse primers used in this work for RT-PCR, ChIP, and bisulfite sequencing.
(0.03 MB DOC)
