# Supplementary material for: Double Strand Breaks Can Initiate Gene Silencing and SIRT1-Dependent Onset of DNA Methylation in an Exogenous Promoter CpG Island
Source: PLoS Genet. 2008 Aug 15;4(8):e1000155. doi: 10.1371/journal.pgen.1000155 (PMC2491723; doi:10.1371/journal.pgen.1000155)
Supplement: Table S1 — Forward and reverse primers used in this work for RT-PCR, ChIP, and bisulfite sequencing. (0.03 MB DOC) [file pgen.1000155.s002.doc]

|  | Fwd | Rev |
| --- | --- | --- |
| HA-SCE RTPCR | TCCTGACTATGCGGGTATGA | TTTACCTTCATCACGACTACGAA |
| GAPDH RTPCR | GAAGGTCGGAGTCAACGGATTT | ATGGGTGGAATCATATTGGAA |
| TK RTPCR and ChIP | CTGCGGGTTTATATAGACGG | CATTGTTATCTGGGCGCT |
| SCE ChIP | CCCTCTCAGTGGCGTCGGAACT | CCCACCCTCTGATGAGTACCT |
| GAPDH ChIP | CAGAGACTGGCTCTTAAAAAGTGC | GTCCACCACCCTGTTGCTGTA |
| Across cut site | GAGGGTCAACGCGTAGGGATAACAGGGTAAT | CCCACCCTCTGATGAGTACCTGCAGCAGCAG |
| SCE bisulfite sequencing | AATAAAAGAATTTAGTTAAGTGT | CCCACCCTCTAATAAATACCT |
